# Supplementary material for: The Impact of Domain Shift on Predicting Perceived Sleep Quality from Wearables
Source: Sensors (Basel). 2025 Jun 27;25(13):4012. doi: 10.3390/s25134012 (PMC12252337; doi:10.3390/s25134012)
Supplement: Supplementary file 1 [file sensors-25-04012-s001.zip › sensors-3604565-supplementary.pdf]

Article

The Impact of Domain Shift on Predicting Perceived Sleep Quality from Wearables

Nouran Abdalazim, Leonardo Alchieri, Lidia Alecci, Pietro Barbiero and Silvia Santini

Faculty of Informatics, Università della Svizzera italiana
* Corresponding author: nouran.abdalazim@usi.ch

In this document, we provide the supplementary materials for the manuscript entitled: *"The Impact of Domain Shift on Predicting Perceived Sleep Quality from Wearables"*. We demonstrate more details regarding: (1) the self-reports included in BiheartS dataset (Supplementary A), (2) the technical details for the features extraction (Supplementary B), (3) the statistical comparison between the two datasets used in our experimental setup, (Supplementary C), (4) the experimental scenarios that we use in the sleep quality recognition task (Supplementary D), (5) the experimental results for the machine learning (ML) models in sleep quality recognition task (Supplementary D), and (6) the hyper-parameter tuning for the proposed approach (Supplementary E).

Supplementary A. Self-reports in BiheartS Dataset

In this subsection, we present an illustration of the collected self-reports in the BiheartS dataset. Table S1 and Table S2 list all the adapted questions used in the morning and evening self-reports respectively, along with the original questions and the names of the validated source questionnaires. For the morning self-reports, we adapt questions from three validated questionnaires, namely: Pittsburgh Sleep Quality Index (PSQI) [1], Daily Use Caregiver Sleep Survey (DUCSS) [2], and recovery scale [3]. Figure S1 shows the pen-paper diary used for the morning self-reports. The questions adopted from these questionnaire target information about sleep behaviour of the previous night. For the evening self-reports, we adapt questions from five questionnaires, namely: Basic Nordic Sleep Questionnaire (BNSQ), Perceived Stress Scale(PSS-10) [4], Fatigue Assessment Scale (FAS), Quality of Life Enjoyment and Satisfaction Questionnaire (Q-LES-Q) [5], and the International Physical Activity Questionnaire (IPAQ) [6]. The questions focus on the daily behaviour that includes the sleepiness during the day, the perception of stress and the tiredness, the general physical health, and the duration of physical activity. Figure S2 shows the pen-paper diary used for the evening self-reports. The questions used in the pen-paper diary are exactly the same as the questions used in the RealLife Exp mobile application. Figure S3 summarizes the data collection protocol for the BiheartS dataset.

 https://doi.org/10.3390/s25134012

Table S1. Summary of all the questions used in the morning self-reports of the BiheartS dataset.

Adapted Question	Original Question	Validated Questionnaire
At what time did you go to bed last night?	During the past month, what time have you usually gone to bed at night?	PSQI [1]
How long (in minutes) has it taken you to fall asleep last night?	During the past month, How long (in minutes) has it usually taken you to fall asleep each night?	PSQI [1]
At what time did you get up in the morning?	During the past month, what time have you usually gotten up in the morning? Usual getting up time	PSQI [1]
How many awakenings did you experience last night?	If you woke up during the night, approximately how long did it take you to fall back to sleep?	DUCSS [2]
If there were any occurrences from last night that disturbed your sleep, please indicate their type and when they occurred.	List any occurrences that you can recall from last night that disturbed your sleep. Please also include the time when they happened.	DUCSS [2]
How well recovered do you feel after your last night's sleep?	This morning, I feel mentally recovered	Recovery scale [3]
During the last night, how would you rate your sleep quality overall?	During the past month, how would you rate your sleep quality overall?	PSQI [1]

Table S2. Summary of all the questions used in the evening self-reports of BiheartS dataset.

Adapted Question	Original Question	Validated Questionnaire
During the current day, how sleepy did you feel overall?	Do you feel excessively sleepy during the daytime ?	BNSQ [7]
During the current day, how nervous/stressed did you feel overall?	In the last month, how often have you felt nervous and stressed?	PSS-10 [4]
During the current day, how tired/fatigued did you feel overall?	• I get tired very quickly<br>• I am bothered by fatigue	FAS [8]
During the current day, how satisfied have you been with your physical health?	During the past week how satisfied have you been with your physical health ?	Q-LES-Q [5]
During the current day, how much time did you spend on physical activities in your leisure time?	• How much time did you usually spend on one of those days doing light physical activities in your leisure time ?<br>• How much time did you usually spend on one of those days doing moderate physical activities in your leisure time ?<br>• How much time did you usually spend on one of those days doing vigorous physical activities in your leisure time ?	IPAQ [6]

	**Today is…**	Date (dd/mm/yyyy): ________________
	At what time did you go to bed last night?	Time (hh:mm): ____________________
	How long (in minutes) has it taken you to fall asleep last night?	___________ minutes
	At what time did you got up in the morning?	Time (hh:mm): ____________________
	How many awakenings did you experience last night?	Number of awakening(s): ___________________<br>Total duration of awakening: ___________ minutes
	If there were any occurrences from last night that disturbed your sleep, please indicate their type and when they occurred. (E.g., feeling too hot/cold, coughing, having nightmares, going to the restroom, etc.)	Type of disturbance(s): ____________________<br>Time(s) when they occurred: ________________
	How well recovered do you feel after your last night's sleep? (0 is not well recovered at all, 10 is very well recovered)	0 ———— 10
	During the last night, how would you rate your sleep quality overall? (0 is very bad, 10 is very good)	0 ———— 10
	Is there anything else about last night's sleep that you would like to mention? (E.g., you slept in a different bed last night)	

Figure S1. Paper-pen morning self-reports used in BiheartS data collection campaign.

	**Today is…**	Date (dd/mm/yyyy): ________________
	During the current day, how sleepy did you feel overall? (0 is not sleepy at all, 10 is very sleepy)	0 ———— 10
	During the current day, how nervous/stressed did you feel overall? (0 is not nervous/stressed at all, 10 is very nervous/stressed)	0 ———— 10
	During the current day, how tired/fatigued did you feel overall? (0 is not tired/fatigued at all, 10 is very tired/fatigued)	0 ———— 10
	During the current day, how satisfied have you been with your physical health?	☐ Very poor ☐ Poor ☐ Fair ☐ Good ☐ Very good
	During the current day, how much time did you spend on physical activities in your leisure time?	Light activities: _____________ minutes (e.g., walking)<br>Moderate activities: __________ minutes (e.g., running at moderate pace)<br>Vigorous activities: ___________ minutes (e.g., running at fast pace)
	Is there anything else about your current day that you would like to mention? (E.g., you felt particularly motivated)	

Figure S2. Paper-pen evening self-reports used in BiheartS data collection campaign.

Supplementary B. Features Extraction From Each Modality

In this subsection, we provide technical details for the extracted features from each modality, the mathematical formulas or the Python library used. Table S3 present the features extracted from the Heart Rate (HR), Skin Temperature (ST), Accelerometer (ACC), and Respiratory Patterns (RP) modalities. Table S4 show the features extracted from the contextual self-reports. Finally, the Table S5 presents the Heart Rate Variability (HRV) features extracted from the Interbeat Interval (IBI) signal.

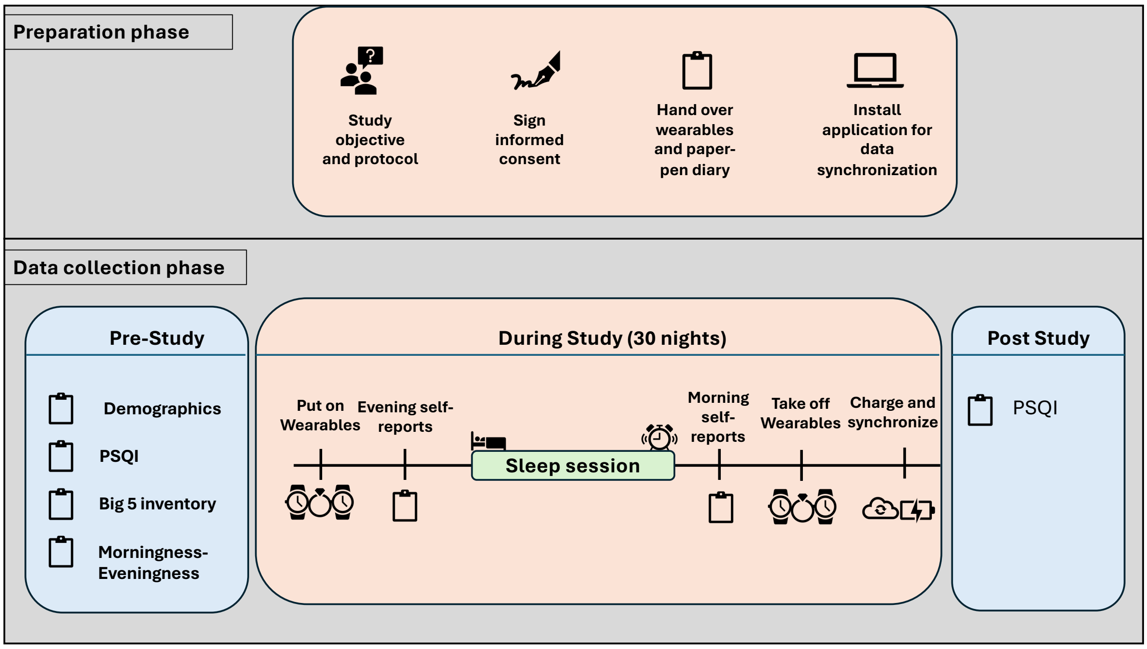

Figure S3. Study protocol for the BiheartS data collection campaign.

Table S3. Summary of the extracted features from HR, ST, ACC, and RP modalities. The features are extracted from each sleep night q as a whole, $q \in \mathbb{R}^n$, where n is the number of sensor 's recording during sleep night. n differs based on the sampling rate of the sensors. $r \in \mathbb{R}^n$ is the signal obtained after applying the Fast Fourier Transform (FFT) as implemented in Scipy [9]. All the features are functions $\mathbb{R}^n \rightarrow \mathbb{R}^1$. In total, we have 36 features: 12 features from HR signal, 12 features from ST signal, 12 features from ACC signal

Feature	Formula
Mean	$\bar{q}$
Median	$median(q)$
Variance	$\sigma^2(q)$
70th Quantile	$Quantile(70) = sorted(q)[0.7 \times n - 1]$
Maximum	$\max(q)$
Minimum	$\min(q)$
Standard error	$\sigma_M = \frac{\sigma}{\sqrt(q)}$
Standard deviation	$\sigma(q)$
Direct current component	$r[0]$
Sum of spectral coefficients	$\sum_{k=1}^{N} \lvert r[k] \rvert$
Information entropy	$-\sum_{k=1}^{N} P(r[k]) \log_2(P(r[k]))$
Energy	$\sum_{k=1}^{N} \lvert r[k] \rvert^2$

Table S4. Summary of the extracted features from self-reports. *bed_time* is the time the user reported going to sleep the previous night, and *wake_time* is the time the user reported getting up in the morning. Both are in the format $hh : mm$. *n_awakening* is the reported number of awakenings during the previous night's sleep. In total, we have 4 contextual features from self-reports.

Feature	Formula
Sleep duration	$wake\_time - bed\_time$ `if` $bed\_time >= 00:00$ `else` $24:00 - bed\_time + wake\_time$
number of awakenings	$n\_awakening$
sleep before mid-night	`True if` $bed\_time < 00:00$ `else False`
wake-up after mid-day	`True if` $wake\_time > 12:00$ `else False`

Table S5. Summary of the extracted features from IBI modality. The features are extracted from each night w as a whole, where $w \in \mathbb{R}^x$, and x varies as it is computed by the Empatica E4 algorithm, which removes incorrect peaks caused by noise in the BVP signal. This makes it impossible to define the number of data points a priori. All the features are functions $\mathbb{R}^n \to \mathbb{R}^1$. The features are extracted using the `hrv-analysis` package. Therefore, we report in the table the functions and the respective features names as they appear in the package, along with a brief description. In total, we have 19 features from IBI signal.

Feature	`hrv-analysis` package	Description
Mean	`get_time_domain_features(w)['mean_nni']`	Mean of IBI
SDNN	`get_time_domain_features(w)['sdnn']`	Standard deviation of IBI
SDSD	`get_time_domain_features(w)['sdsd']`	Standard deviation of differences between adjacent IBI
NN_50	`get_time_domain_features(w)['nni_50']`	Number of interval differences of successive IBI greater than 50 ms
PNNI_50	`get_time_domain_features(w)['pnni_50']`	The proportion derived by dividing NNI_50 by the total number of IBI
NNI_20	`get_time_domain_features(w)['nni_20']`	Number of interval differences of successive IBI greater than 20 ms
PNNI_20	`get_time_domain_features(w)['pnni_20']`	The proportion derived by dividing NNI_20 by the total number of IBI
RMSSD	`get_time_domain_features(w)['rmssd']`	Square root of the mean of the sum of the squares of differences between adjacent IBI
Median	`get_time_domain_features(w)['median_nni']`	Median absolute values of the successive differences between the IBI
Range	`get_time_domain_features(w)['range_nni']`	Difference between the maximum and minimum IBI
CVSD	`get_time_domain_features(w)['cvsd']`	Coefficient of variation of successive differences equal to the RMSSD divided by Mean
CVNNI	`get_time_domain_features(w)['cvnni']`	Coefficient of variation equal to the ratio of SDNN divided by Mean
LF	`get_frequency_domain_features(w)['lf']`	Variance in HRV in the low frequency (0.04-0.15 Hz)
HF	`get_frequency_domain_features(w)['hf']`	Variance in HRV in the high frequency (0.15-0.40 Hz)
VLF	`get_frequency_domain_features(w)['vlf']`	Variance in HRV in the very low frequency (0.003-0.04 Hz)
LF/HF Ratio	`get_frequency_domain_features(w)['lf_hf_ratio']`	Ratio between LF and HF
$LF_{normalized}$	`get_frequency_domain_features(w)['lfnu']`	Normalized LF power
$HF_{normalized}$	`get_frequency_domain_features(w)['hfnu']`	Normalized HF power
Total Power	`get_frequency_domain_features(w)['total_power']`	Total power density spectral

Supplementary C. Statistical Comparison Between Datasets

In this subsection, we present the results for the statistical comparison between the M2sleep and the BiheartS datasets. To investigate the existence of covariate shift between the BiheartS and the M2sleep datasets, we compute Spearman's rank correlation coefficient between all the extracted features and the reported sleep quality label. In Figure S4, we report the obtained correlation coefficient for each feature from the HR, ST, ACC, RP and contextual modalities in each dataset. In Figure S5 shows the correlation results of the

features from the HRV modality. The results show that the two datasets have different correlation patterns. This implies that the distribution of the features differs between the BiheartS and the M2sleep datasets, i.e., existence of covariate shift.

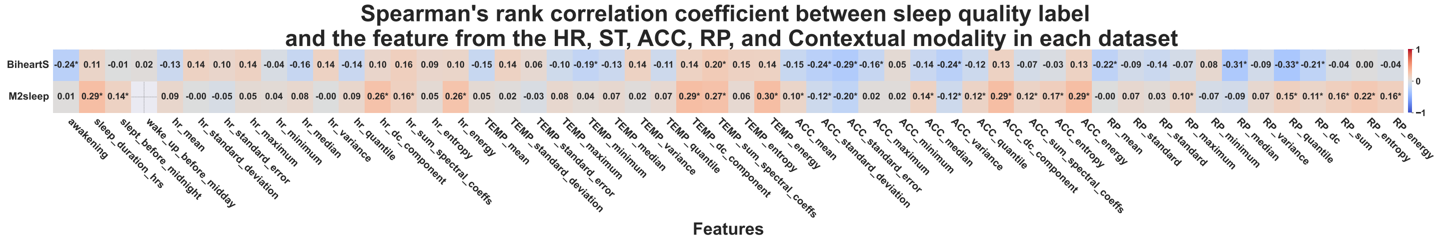

Figure S4. Results of Spearman's rank correlation coefficient between the features extracted from the HR, ST, ACC, RP and contextual modalities and the reported sleep quality. (*) symbol indicate that the correlation value is statistically significant with $\alpha = 0.05$.

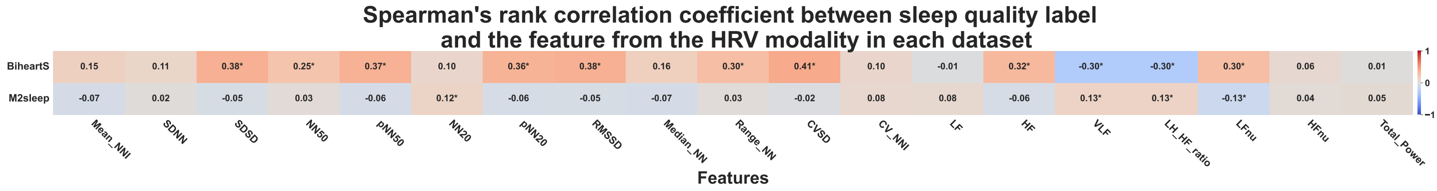

Figure S5. Results of Spearman's rank correlation coefficient between the features extracted from the HRV, modality and the reported sleep quality. (*) symbol indicate that the correlation value is statistically significant with $\alpha = 0.05$.

Supplementary D. Impact of Domain Shift on Sleep Quality Recognition

In this subsection, we present more details regarding the three scenarios used to evaluate the ML models for sleep quality recognition task as well as the results of the ML models in this classification task.

Supplementary D.1. Evaluation Scenarios and Evaluation Metrics

As mentioned before, we use a sleep quality recognition task to assess the impact of covariate shift on population and personalized models. To this end, we rely on three distinct setups, hereafter *scenarios*, to train and validate our models. In the *Single-dataset scenario*, we train and validate the models using each dataset independently. This scenario is similar to those used in the literature, where researchers train and validate their models only on a single dataset. On the other hand, in the *multiple-dataset scenario* we train our models using data from one dataset, e.g., BiHeartS, and then test it only using data from the other dataset, e.g., M2Sleep. We use this scenario to simulate a real-world application, where a model is trained on a specific set of data and then applied on a different, in time, users and space, dataset. Finally, in the *mixed-datasets scenario* we combine the two datasets, M2Sleep and BiHeartS, into a single dataset.

In the *Single-dataset* and *Mixed datasets* scenarios, we report our results using the Leave-One-Participant-Out (LOPO) cross validation method. In this validation method, for each iteration we train the model using data from all users but one, which is left out for testing. The number of cross validation iterations corresponds to the number of users. In the other hand, the *multiple-dataset scenario* cioncides with a Leave-One-Dataset-Out validation method, since the train set is always from one dataset and the test set always from the other.

Accordingly, we formulate the single-dataset scenario as follows: A dataset D is available for the training and evaluation of the ML model. $D = \{(x_i, y_i)\}_{i=1}^{N_D}$, where N_D is the number of records in dataset D. x_i is the $i-$th feature vector in the dataset D.

$x_i \in \mathbb{R}^l$, where l is the number of features depending on the modalities. y_i is the label for x_i, and $y_i \in [0,1]$. Let $U_D = \{u_1, u_2, ..., u_m\}$, U_D be the set of unique users in dataset D. $D_u = \{(x_j, y_j)\}_{j=1}^{N_u}$, where D_u is the set of labeled data points collected from user u, N_u is the number of data points collected from user u. For training and evaluation, we hold-out one user for the evaluation. Accordingly, $\forall u \in U_D$, the $training\_set = D - D_u$ and the $testing\_set = D_u$. With this scenario, there is no user-data leakage, since the target test user is never present in the train set. However, in this scenario "dataset-specific" traits could still be present in both the train and test sets.

Moreover for the multiple-dataset scenario, we formulate this scenario as follows: assume two datasets D_1 and D_2 are available for the training and evaluation of the ML model. $D_z = \{(x_{zi}, y_{zi})\}_{i=1}^{N_{D_z}}$, where N_{D_z} is the number of records in dataset D_z and $z \in [1,2]$. x_{zi} is a the $i-$th feature vector in the dataset D_z. $x_{zi} \in \mathbb{R}^l$, where l is the number of features depending on the modalities. y_{zi} is the label for x_{zi}, and $y_{zi} \in [0,1]$. Let $U_{D_z} = \{u_{z1}, u_{z2}, ..., u_{zm}\}$ be the set of unique users in dataset D_z. $D_{u_z} = \{(x_j, y_j)\}_{j=1}^{N_u}$, where D_{u_z} is the set of labeled data points in dataset z collected from user u, N_u is the number of data points collected from user u. For training, we use the records from all users U_{D_1} in D_1, the $training\_set = D_1$. Then, the trained model is evaluated using data from each user in the other dataset D_2. Accordingly, $\forall u \in U_{D_2}$, the $testing\_set = D_u$.

Finally, we formulate the mixed-datasets scenario as follows: assume two datasets D_1 and D_2 are available for the training and evaluation of the ML model. $D_z = \{(x_{zi}, y_{zi})\}_{i=1}^{N_{D_z}}$, where N_{D_z} is the number of records in dataset D_z and $z \in [1,2]$. x_{zi} is a $i-$th feature vector in the dataset D_z. $x_{zi} \in \mathbb{R}^l$, where l is the number of features depending on the modalities. y_{zi} is the label for x_{zi}, and $y_{zi} \in [0,1]$. Let $U_{D_z} = \{u_{z1}, u_{z2}, ..., u_{zm}\}$, denote the set of unique users in dataset D_z. $D_{u_z} = \{(x_j, y_j)\}_{j=1}^{N_u}$, where D_u is the set of labeled data points in dataset z collected from user u, N_u is the number of data points collected from user u. For training and evaluation, we merge the two datasets D_1 and D_2 in one single dataset D, where $D = D_1 \cup D_2$ and $U = U_{D_1} \cup U_{D_2}$. Then we hold-out one user for the evaluation . Accordingly, $\forall u \in U$, the $training\_set = D - D_u$ and the $testing\_set = D_u$.

For the evaluation metrics, the prequential accuracy (PreAcc) metric is typically used for evaluating the performance of online models over time, as suggested in [10]. The computation of the PreAcc is defined in Equation S1.

$$\text{PreAcc}(t) = \begin{cases} acc_{ex}(t), & \text{if } t = t_0 \\ \text{acc}(t-1) + \dfrac{acc_{ex}(t) - \text{acc}(t-1)}{t - t_0 + 1}, & \text{otherwise.} \end{cases} \tag{S1}$$

Where $acc_{ex} = 1$, if the data point is correctly classified, otherwise $acc_{ex} = 0$, and t_0 is the initial step for evaluation. In our experimental setup, we assume that the target test user provides a feedback for each inference result. Accordingly, the t_0 in Equation S1 is equals to 1 and the t Equation S1 is equals to the total number of data points available from the target test user. Consequently, the output PreAcc metric is mathematically equivalent to the traditional accuracy metric. Accordingly, we rely on the accuracy metric to evaluate both the online models and the offline models in our experiments.

Supplementary D.2. Experimental Results for Sleep Quality Recognition

To investigate the impact of the domain shift in particular covariate shift on the performance on the personalized model and the population model, we evaluate the performance of these models in three different scenarios using different modalities. Also, we implement both models using the Passive Aggressive Classifier (PAC) [11] and the Multilayer Preceptron Classifier (MLP) [12,13]. The PAC classifier is often used in the online learning settings [11], and the MLP classifier is widely used in sleep behaviour analysis in the literature, e.g., [14–16].

We present in Table S6 and Table S7, the results for the binary sleep quality recognition task. We present the results for the population (offline) models and the personalized (online) models, for both the PAC and MLP classifiers and the two datasets, BiHeartS and M2sleep, separately. We also report the results of the baseline classifiers, and we indicate whether our models achieve a statistically higher accuracy (when using a Wilcoxon statistical test) than the two baselines. We report the results when using the single modality and the multi-modality feature sets.

Our results show that in the single-dataset scenario, the personalized models and the population models have a comparable performance in most of the modalities using either the PAC or the MLP classifiers. However, when using two different datasets for models training and evaluation (multiple-dataset scenario), the personalized models outperform the population models. Moreover, there is a significant drop in the performance of the population model in the multiple-dataset scenario, while the performance of the personalized model is not affected with respect to the single-dataset scenario. Finally, for the mixed-datasets scenario, we observe that personalized models achieve a higher performance compared to the population models in all the modalities using the PAC classifier, and most of the modalities using the MLP classifier.

Based on the results in Table S6 and Table S7, we compare the performance of the personalized models and the population models across the single, the multiple datasets, and the mixed-datasets scenarios. This analysis shows the impact of using different datasets for training and evaluation on the performance of both models.

Table S6. Results of the sleep quality recognition binary task for single and multiple-dataset scenarios. We report the mean accuracy (%)±standard error. Symbols indicate statistical significance of the models: (*) vs. random guess, (**) vs. random and biased random guess, (†) vs. biased random guess. Bold values show significant differences between population and personalized models within the same modality and scenario. All statistical comparisons are done using the Wilcoxon paired non-parametric test ($\alpha = 0.05$).

Passive Aggressive Classifier Performance (test on the M2sleep dataset)

**Modality**	**Single-dataset**		**Multiple-dataset**		**Mixed-dataset**	
	Population	Personalized	Population	Personalized	Population	Personalized
HR	54.03±0.01 *	**62.28±0.00** **	48.7 ± 0.01	**60.17±0.00** **	52.53 ± 0.01 *	**62.31 ± 0.01** **
HRV	50.98±0.01 *	**62.33 ± 0.00** **	49.33±0.01 †	**61.64 ± 0.00** **	51.69 ± 0.01 *	**61.7 ± 0.00** **
ST	50.42±0.01 *	**62.90±0.01** **	51.90±0.01 **	**63.25±0.01** **	53.31 ± 0.01 *	**64.74±0.01** **
ACC	53.24±0.01 **	**61.12±0.00** **	54.06±0.01 **	**61.92±0.00** **	54.54 ± 0.01 **	**62.18 ± 0.00** **
RP	54.3 ± 0.01*	**63.92 ± 0.00****	51.69 ± 0.01**	**62.92 ± 0.0****	54.36 ± 0.01 †	**64.18 ± 0.00****
All-sensor-features	53.06 ± 0.01 *	**63.40±0.0** **	53.06 ± 0.01 **	**62.97 ± 0.0** **	51.94 ± 0.01 *	**63.34 ± 0.00** **
Contextual	56.57±0.01 **	**64.48±0.01** **	48.50 ± 0.01 †	**64.12 ± 0.00** **	53.32 ± 0.01 *	**64.55 ± 0.01** **
All-features	50.96±0.01 *	**63.75±0.00** **	52.41 ± 0.01 **	**63.55 ± 0.00** **	52.0 ± 0.01 *	**63.67 ± 0.00** **
BRG baseline	53.48±0.01	51.67±0.00	46.93±0.01	49.24±0.00	52.64±0.01	51.27±0.00
RG baseline	47.44±0.01					

Passive Aggressive Classifier Performance (test on the BiheartS dataset)

**Modality**	**Single-dataset**		**Multiple-dataset**		**Mixed-dataset**	
	Population	Personalized	Population	Personalized	Population	Personalized
HR	50.22 ± 0.01	**66.96 ± 0.01** **	47.93±0.01	**63.34 ± 0.01** **	49.99 ± 0.01 †	**64.87 ± 0.01** **
HRV	62.28±0.01 **	65.17±0.01 **	45.60±0.01	**64.30±0.01** **	50.0 ± 0.01 †	**64.72±0.01** **
ST	52.61 ± 0.01	**64.92±0.01** **	50.38±0.01 †	**61.32±0.01** **	50.81 ± 0.01 †	**62.58 ± 0.01** **
ACC	49.62 ± 0.01	**61.20 ± 0.01** **	49.56±0.01 †	**60.83±0.01** **	50.02 ± 0.01 †	**60.39 ± 0.01** **
RP	57.97 ± 0.01**	**65.82 ± 0.01****	46.82 ± 0.01	**66.88 ± 0.01****	50.91 ± 0.01 †	**65.68 ± 0.01****
All-sensor-features	64.41** ± 0.01 **	**69.85 ± 0.01** **	49.63±0.01 †	**62.77±0.00** **	55.99 ± 0.01 †	**64.9 ± 0.01** **
Contextual	48.27 ± 0.01	**67.12 ± 0.01** **	48.63±0.01	**65.66±0.01** **	53.41 ± 0.01 †	**64.92 ± 0.01** **
All-features	62.02 ± 0.01 **	**70.39 ± 0.01** **	49.02 ± 0.01 †	**64.52 ± 0.01** **	53.13±0.01 †	**65.5 ± 0.01** **
BRG baseline	51.60 ± 0.01	51.12 ± 0.01	46.06 ± 0.01	47.51 ± 0.01	46.39±0.01	48.41±0.01
RG baseline	52.81±0.01					

Table S7. Results of the sleep quality recognition binary task for single and multiple-dataset scenarios. We report the mean accuracy (%)±standard error. Symbols indicate statistical significance of the models: (*) vs. random guess, (**) vs. random and biased random guess, (†) vs. biased random guess. Bold values show significant differences between population and personalized models within the same modality and scenario. All statistical comparisons are done using the Wilcoxon paired non-parametric test ($\alpha = 0.05$).

Multilayer Perceptron Classifier Performance (test on the M2sleep dataset)

Modality	Single-dataset		Multiple-dataset		Mixed-dataset	
	Population	Personalized	Population	Personalized	Population	Personalized
HR	59.80±0.01 **	60.25±0.01 **	46.94 ± 0.01	**58.19 ± 0.01** **	60.36±0.01 **	60.50±0.01 **
HRV	52.01±0.01 *	**62.28±0.01** **	46.75 ± 0.01 †	**60.91 ± 0.01** **	51.16 ± 0.01 *	**62.52 ± 0.01** **
ST	**61.55±0.01** **	59.63±0.01 **	54.81 ± 0.01 **	**59.03 ± 0.01** **	56.27 ± 0.00 **	**60.07±0.01** **
ACC	**60.93±0.01** **	59.21±0.01 **	55.84 ± 0.00 **	**57.26 ± 0.01** **	58.33±0.01 **	59.33±0.01 **
RP	60.33 ± 0.01**	59.94 ± 0.01**	53.75 ± 0.01**	**57.5 ± 0.01****	56.25 ± 0.01*	**59.55 ± 0.01****
All-sensor-features	55.53 ± 0.01 *	**63.74 ± 0.01** **	57.4 ± 0.01 **	**62.37 ± 0.01** **	52.84 ± 0.01 *	**63.66 ± 0.01** **
Contextual	60.69±0.01 **	60.42±0.01 **	43.99 ± 0.01	**59.96 ± 0.01** **	60.39±0.01 **	61.30±0.01 **
All-features	54.83 ± 0.01 *	**64.95 ± 0.01** **	56.55 ± 0.01 **	**62.97 ± 0.01** **	53.19 ± 0.01 *	**64.35 ± 0.01** **
BRG Baseline	53.48±0.01	51.67±0.00	46.93±0.01	49.24±0.0	52.53 ± 0.01	51.25 ± 0.0
RG Baseline			47.44±0.01			

Multilayer Perceptron Classifier Performance (test on the BiheartS dataset)

Modality	Single-dataset		Multiple-dataset		Mixed-dataset	
	Population	Personalized	Population	Personalized	Population	Personalized
HR	58.62 ± 0.01 **	57.38 ± 0.01 **	46.58±0.01	**53.31±0.01** †	45.31±0.01	**53.24±0.01** †
HRV	**64.77 ± 0.01** **	59.6 ± 0.01 **	41.87 ± 0.01	**52.7 ± 0.01** †	53.67±0.01 †	54.79±0.01 †
ST	**59.23±0.01** **	55.17±0.01 †	47.10 ± 0.01 †	52.56±0.01 †	53.30±0.01 †	54.49±0.01 †
ACC	57.02 ± 0.01**	56.91 ± 0.01 †	47.33 ± 0.01 †	**553.72 ± 0.01** †	51.58±0.01 †	**54.57±0.01** †
RP	**62.21 ± 0.01****	56.68 ± 0.01 †	38.41 ± 0.01	**53.88 ± 0.01**†	53.68 ± 0.01 †	**55.36 ± 0.01**†
All-sensor-features	**63.73 ± 0.01****	59.03 ± 0.01 †	45.19 ± 0.01	**56.48 ± 0.01** †	49.92 ± 0.01 †	**57.45±0.01** †
Contextual	51.17±0.01	52.26±0.01	48.89 ± 0.01 †	**51.32 ± 0.01** †	43.16±0.01	**50.88±0.01** †
All-features	59.85 ± 0.01 **	**61.28 ± 0.01****	43.21 ± 0.01	**55.39 ± 0.01** †	45.64 ± 0.01 †	**56.36 ± 0.01** †
BRG Baseline	51.60 ± 0.01	51.12 ± 0.01	46.06 ± 0.01	47.51 ± 0.01	46.38±0.01	48.61 ± 0.01
RG Baseline			52.81±0.01			

Supplementary E. Hyper-parameters for HDBSCAN Clustering Algorithm

We rely on the unsupervised domain adaptation approach to mitigate the impact of the covariate shift between different datasets. We propose a cluster-based population model approach that integrates the Hierarchical Density-Based Spatial Clustering of Applications with Noise (HDBSCAN) clustering algorithm with the population model.

We run an experiment to select the *min_cluster_density* and the *min_samples* hyper-parameters for the HDBSCAN algorithm. We explore different values for each parameter ranging from $[2, 50]$. We conduct this experiment for each modality in the M2sleep and the BiheartS datasets separately. We use the Silhouette Coefficient as a metric of the quality of the obtained clusters. For each distinct combination of the two hyper-parameters, we compute the Silhouette Coefficient for the obtained clusters and we count the number of data points that are labeled as noise by the HDBSCAN algorithm.

Figure S7 and Figure S6 show the distribution of the Silhouette Coefficient with the different values of the *min_cluster_density* and the *min_samples* hyper-parameters. We choose the values for the *min_cluster_density* and the *min_samples* hyper-parameters that achieve the highest Silhouette Coefficient with the minimum number of data points labeled as noise by HDBSCAN algorithm.

Table S8 shows the selected values of the *min_cluster_size* and *min_samples* hyper-parameters for each modality in each dataset. Also in Table S8, we present the percentage of the feature vectors in each modality that are labelled as noise as well as the values of the two hyper-parameters that achieve the highest Silhouette Coefficient. We choose the values for the *min_cluster_density* and the *min_samples* hyper-parameters that achieve the

highest Silhouette Coefficient with the minimum number of data points labelled as noise by the HDBSCAN algorithm.

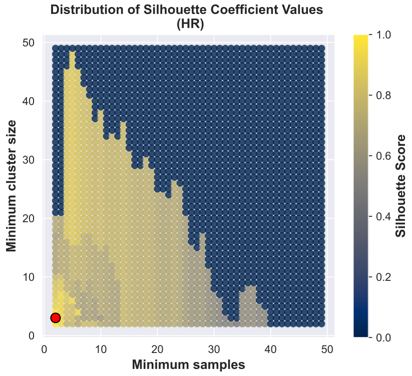

(a) Heart rate modality.

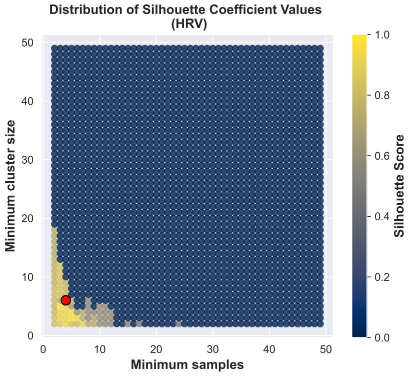

(b) Heart rate variability modality.

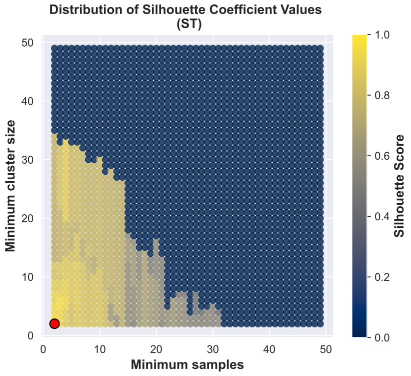

(c) Skin temperature modality.

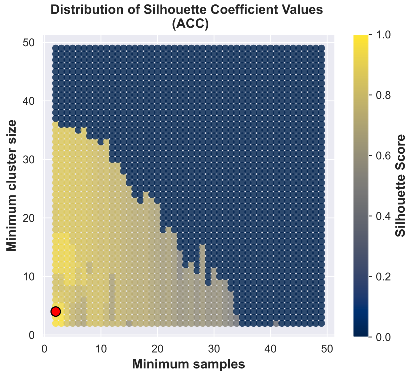

(d) Accelerometer modality modality.

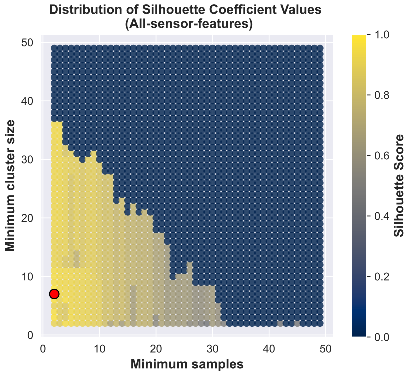

(e) All-sensor-features modality.

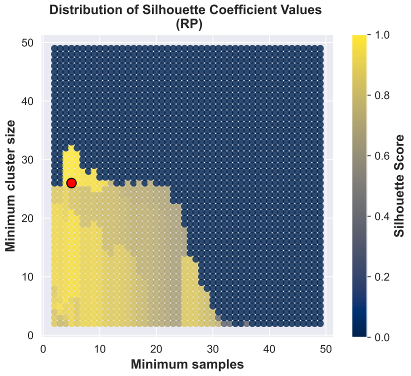

(f) Respiratory Pattern modality.

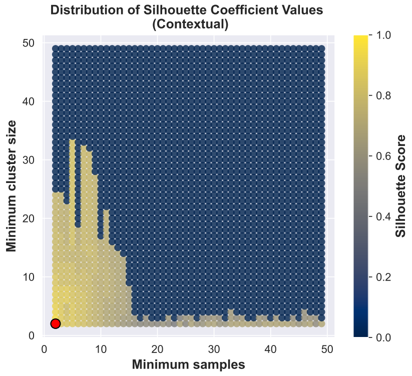

(g) Contextual modality.

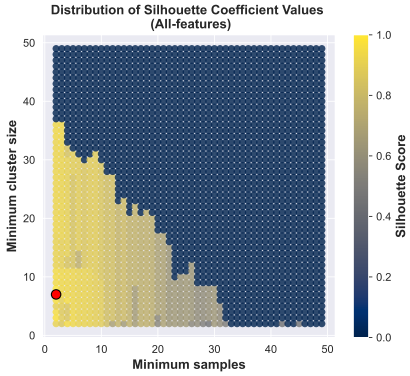

(h) All-features modality.

Figure S6. Illustration of the Silhouette Coefficient distribution for clustering the BiheartS dataset using the HDBSCAN algorithm with varying values of the *min_samples* and *min_cluster_density* hyper-parameters. The red circle represents the highest Silhouette Coefficient value obtained.

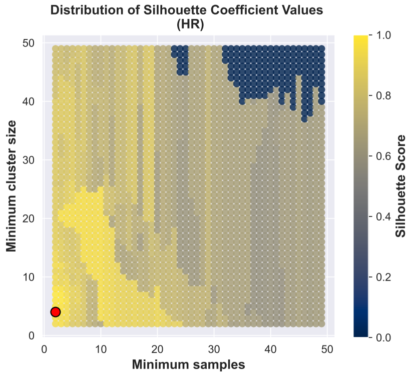

(a) Heart rate modality.

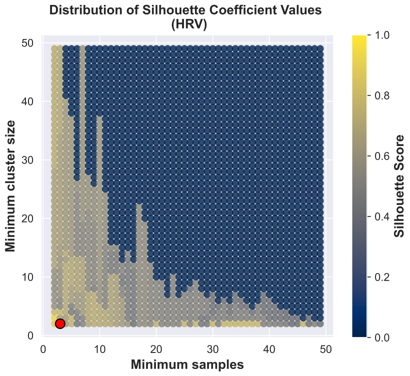

(b) Heart rate variability modality.

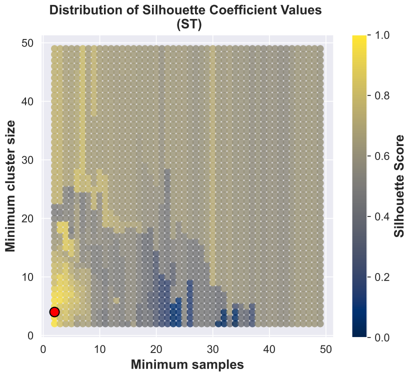

(c) Skin temperature modality.

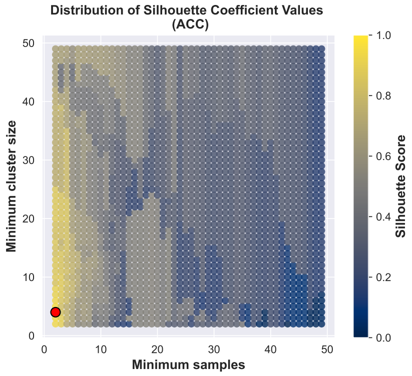

(d) Accelerometer modality.

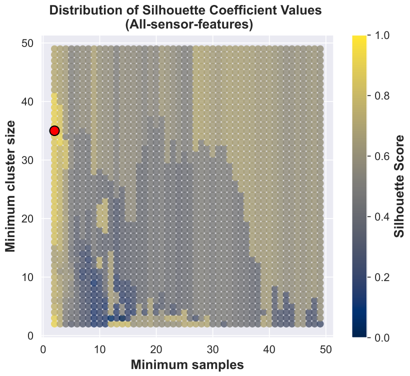

(e) All-sensors-features modality.

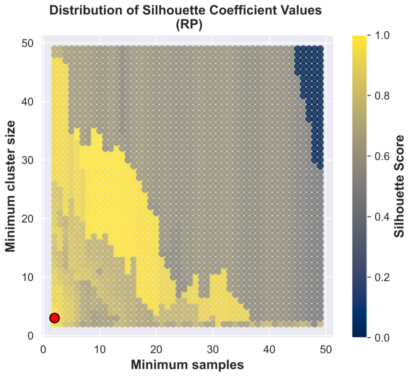

(f) Respiratory Pattern modality.

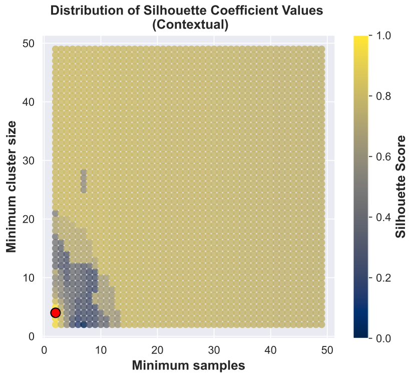

(g) Contextual modality.

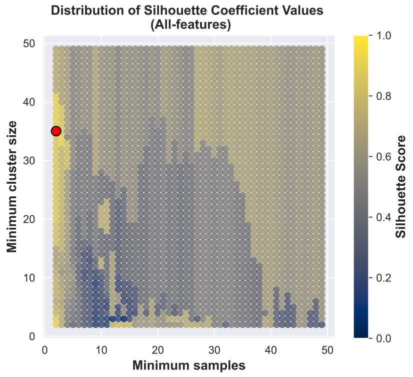

(h) All-features modality.

Figure S7. Illustration of the Silhouette Coefficient distribution for clustering the M2sleep dataset using the HDBSCAN algorithm with varying values of the *min_samples* and *min_cluster_density* hyper-parameters. The red circle represents the highest Silhouette Coefficient value obtained.

Table S8. Illustration of the selected values for the *min_cluster_size* and *min_samples* hyper-parameters in the HDBSCAN clustering algorithm, determined based on the highest Silhouette Coefficient for each modality within each dataset. The noise ( ) indicates the percentage of feature vectors in the dataset that do not belong to any of the formed clusters.

Modality	Silhouette Coefficient	*min_cluster_size*	*min_samples*	Noise (%)
**M2sleep dataset**				
HR	0.48	4	2	9
HRV	0.45	2	3	2
ST	0.42	4	2	11
ACC	0.50	4	2	9
RP	0.52	3	2	7
All-sensor-features	0.27	35	2	19
Contextual	0.45	4	2	14
All-features	0.27	35	2	19
**BiheartS dataset**				
HR	0.49	2	3	12
HRV	0.25	6	4	15
ST	0.49	2	2	11
ACC	0.54	4	2	7
All-sensor-features	0.37	7	2	16
Contextual	0.36	2	2	19
All-features	0.37	7	2	16

References

1. Buysse, D.J.; Reynolds III, C.F.; Monk, T.H.; Berman, S.R.; Kupfer, D.J. The Pittsburgh Sleep Quality Index: a new instrument for psychiatric practice and research. *Psychiatry research* **1989**, *28*, 193–213.
2. Sadeghi, R.; Banerjee, T.; Hughes, J.C.; Lawhorne, L.W. Sleep quality prediction in caregivers using physiological signals. *Computers in biology and medicine* **2019**, *110*, 276–288.
3. Hur, W.M.; Shin, Y. Is resting and sleeping well helpful to job crafting? Daily relationship between recovery experiences, sleep quality, feelings of recovery, and job crafting. *Applied Psychology* **2023**, *72*, 1608–1623.
4. Cohen, S. Perceived stress in a probability sample of the United States. *The social psychology of health/Sage* **1988**.
5. Endicott, J.; Nee, J.; Harrison, W.; Blumenthal, R. Quality of Life Enjoyment and Satisfaction Questionnaire: a new measure. *Psychopharmacology bulletin* **1993**, *29*, 321–326.
6. Craig, C.L.; Marshall, A.L.; Sjöström, M.; Bauman, A.E.; Booth, M.L.; Ainsworth, B.E.; Pratt, M.; Ekelund, U.; Yngve, A.; Sallis, J.F.; et al. International physical activity questionnaire: 12-country reliability and validity. *Medicine & science in sports & exercise* **2003**, *35*, 1381–1395.
7. Partinen, M.; Gislason, T. Basic Nordic Sleep Questionnaire (BNSQ): a quantitated measure of subjective sleep complaints. *Journal of sleep research* **1995**, *4*, 150–155.
8. Michielsen, H.J.; De Vries, J.; Van Heck, G.L. Psychometric qualities of a brief self-rated fatigue measure: The Fatigue Assessment Scale. *Journal of psychosomatic research* **2003**, *54*, 345–352.
9. Virtanen, P.; Gommers, R.; Oliphant, T.E.; Haberland, M.; Reddy, T.; Cournapeau, D.; Burovski, E.; Peterson, P.; Weckesser, W.; Bright, J.; et al. SciPy 1.0: Fundamental Algorithms for Scientific Computing in Python. *Nature Methods* **2020**, *17*, 261–272. https://doi.org/10.1038/s41592-019-0686-2.
10. Hidalgo, J.I.G.; Maciel, B.I.; Barros, R.S. Experimenting with prequential variations for data stream learning evaluation. *Computational Intelligence* **2019**, *35*, 670–692.
11. Crammer, K.; Dekel, O.; Keshet, J.; Shalev-Shwartz, S.; Singer, Y.; Warmuth, M.K. Online passive-aggressive algorithms. *Journal of Machine Learning Research* **2006**, *7*.
12. Popescu, M.C.; Balas, V.E.; Perescu-Popescu, L.; Mastorakis, N. Multilayer perceptron and neural networks. *WSEAS Transactions on Circuits and Systems* **2009**, *8*, 579–588.
13. Hornik, K.; Stinchcombe, M.; White, H. Multilayer feedforward networks are universal approximators. *Neural networks* **1989**, *2*, 359–366.
14. Sathyanarayana, A.; Joty, S.; Fernandez-Luque, L.; Ofli, F.; Srivastava, J.; Elmagarmid, A.; Arora, T.; Taheri, S.; et al. Sleep quality prediction from wearable data using deep learning. *JMIR mHealth and uHealth* **2016**, *4*, e6562.
15. Yeckle, J.; Manian, V. Automated Sleep Stage Classification in Home Environments: An Evaluation of Seven Deep Neural Network Architectures. *Sensors* **2023**, *23*, 8942.
16. Arora, A.; Chakraborty, P.; Bhatia, M. Analysis of data from wearable sensors for sleep quality estimation and prediction using deep learning. *Arabian Journal for Science and Engineering* **2020**, *45*, 10793–10812.
